# *Ackermannviridae* bacteriophage against carbapenem-resistant *Klebsiella pneumoniae* of capsular type 64

**DOI:** 10.3389/fmicb.2024.1462459

**Published:** 2024-09-23

**Authors:** Juan Li, Yu Feng, Huan Luo, Qingqing Fang, Yongqiang Yang, Zhiyong Zong

**Affiliations:** ^1^Center of Infectious Diseases, West China Hospital, Sichuan University, Chengdu, China; ^2^Division of Infectious Diseases, State Key Laboratory of Biotherapy, Chengdu, China; ^3^Laboratory of Pathogen Research, West China Hospital, Sichuan University, Chengdu, China; ^4^Department of General Practice, General Practice Medical Center, West China Hospital, Sichuan University, Chengdu, China; ^5^State Key Laboratory of Respiratory Health and Multimorbidity, Chengdu, China

**Keywords:** antimicrobial resistance, phage therapy, *Klebsiella pneumoniae*, microbiology, bacteriophages, phage biology

## Abstract

Lytic bacteriophages (phages) are promising clinically viable therapeutic options against carbapenem-resistant *Klebsiella pneumoniae* (CRKP). In China, the predominant strains are those assigned to sequence type 11 and capsular type 64 (ST11-KL64). The emergence of phage resistance is a major bottleneck hindering effective phage therapy, requiring more new phages to provide the flexibility for creating different phage cocktails. However, the majority of phages against ST11-KL64 CRKP belong to the genus *Przondovirus* of the family *Autographiviridae*, which limits the options for constructing cocktails. We recovered a novel lytic phage of the genus *Taipeivirus* within the family *Ackermannviridae* against ST11-KL64 CRKP from a river in China. We phenotypically characterized this phage and obtained its genome sequence for analysis. This phage can inhibit the growth of ST11-KL64 CRKP for 6.5 h at a 0.1 multiplicity of infection and exhibits a narrow host range, being unable to attack CRKP strains of the other 30 capsular types. This phage carries no genes encoding antimicrobial resistance, virulence, or lysogeny. It is stable across a wide range of temperatures and pH values, making it suitable for phage therapy. Unlike other *Taipeivirus* phages, P01 has two tail spike proteins and a unique tail fiber protein. The distinct tail composition of this phage contributes to its activity against ST11-KL64 CRKP and its narrow host range. Taken together, we recovered a phage of a novel viral species with the potential for therapy, which expands the phage biobank against CRKP.

## Introduction

Antimicrobial resistance in clinically significant bacteria such as carbapenem-resistant *Klebsiella pneumoniae* (CRKP), has emerged as a severe global problem (World Health Organization, 2024). The limited pipeline in the development of new antimicrobial agents indicates the urgent need for alternative therapies. Bacteriophages (phages) are viruses that attack bacteria, and some phages can lyse bacteria and can be used for treating bacterial infections (Oechslin, 2018; Nikolich and Filippov, 2020). However, bacteria commonly develop resistance to phages after exposure. One strategy for countering phage resistance is to obtain more phages against the target bacteria to constructing different phage cocktails (Labrie et al., 2010; Tkhilaishvili et al., 2019; Wang et al., 2021). *K. pneumoniae* of sequence type 11 and capsular type 64 (ST11-KL64) is the predominant type of CRKP in China (Zhou et al., 2020). Lytic phages against ST11-KL64 CRKP typically belong to the genus *Przondovirus* of the family *Autographiviridae* within the order *Caudovirales* (Fang et al., 2022; Yin and Zong, 2022). We have previously demonstrated that the combination of multiple phages belonging to different species of the genus *Przondovirus* does not prolong the inhibition of the emerging phage resistance, which is likely due to the competition for the same receptor (Yin and Zong, 2023). Therefore, new phages belonging to different genera or families are needed to expand the phage biobank against CRKP. Here, we report a new phage belonging to the genus *Taipeivirus* of the family *Ackermannviridae* that effectively lyses ST11-KL64 CRKP.

## Methods

### Bacterial strain, genome sequencing, and *in vitro* susceptibility testing

We used a clinical CRKP strain, 135080, for the study and subjected it to whole-genome sequencing using the HiSeq X10 system (Illumina, San Diego, CA). We assembled reads into contigs using SPAdes v3.15.3 (Bankevich et al., 2012) in isolate mode. We performed precise species identification based on average nucleotide identity (ANI) using JSpeciesWS based on BLAST (Peplies et al., 2016) with a ≥ 95% cutoff to define a bacterial species (Richter and Rossello-Mora, 2009). We assigned the strain to a sequence type (ST) using MLST 2.0 (Larsen et al., 2012) by querying the PubMLST database^1^ and determined the capsular type using Kleborate v2.2.0 (Lam et al., 2021). We screened for *β*-lactamase encoding genes using AMRFinderPlus v3.10.23 (Feldgarden et al., 2021). We determined minimum inhibitory concentrations (MICs) of meropenem and imipenem using the Clinical and Laboratory Standards Institute (CLSI) microdilution method (CLSI, 2022).

### Phage isolation

After we determined that strain 135080 was an ST11-KL64 CRKP (see Results section), we used it for phage isolation. We obtained a 200-mL water sample from a river in Shanghai in May 2022. We filtered the water sample through a 0.22-μm membrane and mixed 17 mL of filtered sewage, 2 mL 10× LB broth, and 1 mL overnight culture of strain 135080 for incubation at 37°C for 4 h. We centrifuged the co-culture at 12,000×*g* at 4°C for 2 min, filtered the supernatant through a 0.22-μm membrane, and used Tris–HCl-MgSO_4_ (TM buffer) to dilute the supernatant. We obtained individual plaques by tenfold dilution of the supernatant against strain 135080 using the double-layer agar method as described previously (Green and Sambrook, 2014; Chen et al., 2019). We further purified the isolated phage plaque five times to ensure single phage purification. We therefore obtained a phage capable of lysing 135080 and named it P01 (also called PH1).

### Phage genome sequencing and taxonomic assignment

We prepared the genomic DNA of phage P01 using a Phage DNA isolation Kit (Norgen Biotek; Thorold, Canada) following the manufacturer’s protocol. The preparation included treatment with DNase I and RNase to remove nucleic acids from host bacterial cells when preparing phage particles, and another round of DNase I treatment before lysing the intact phages using proteinase K. We sequenced the genome of P01 using the HiSeq X10 platform. Given the large quantity of sequencing data obtained (see Results section), we minimized host genome contamination by randomly subsampling 1,000,000 reads. This provided approximately 940× coverage of the target phage, which was then used for genome assembly using Unicycler v0.5.0 (Wick et al., 2017). We also checked for contamination and discarded contigs that did not belong to the phage using CheckV (Nayfach et al., 2021). We annotated the phage genome using pharokka v1.7.2 (Bouras et al., 2023) and phold v0.1.4 (https://github.com/gbouras13/phold). We predicted the lifestyle of P01 using PhaTYP (Shang et al., 2023). We searched for the phage sharing the highest overall DNA similarity (identity × coverage) with P01 using BLAST.^2^ We determined the taxonomy of P01 according to the rule defined by the International Committee on Taxonomy of Viruses (ICTV). For the genus *Taipeivirus*, species demarcation is ≥95% overall DNA similarity.^3^ In addition to overall DNA similarity, we also generated a heatmap of mutual intergenomic similarities among *Taipeivirus* phages using VIRIDIC (Moraru et al., 2020). We also performed a maximum-likelihood phylogenetic analysis based on the amino acid sequences of two critical viral components, i.e., the DNA polymerase and the major capsid protein, for all *Taipeivirus* phages (*n* = 23) following the recommendation of the ICTV.

### Comparison with other *Taipeivirus* phages

For further exploration of the genetic features of P01, we retrieved all complete assemblies of *Taipeivirus* (*n* = 23) from the NCBI GenBank collection (accessed 13 June 2024). We annotated these genomes using pharokka v1.7.2 (Bouras et al., 2023) and phold v0.1.4.^4^ We clustered orthologous genes using PIRATE v1.0.5 (Bayliss et al., 2019), aligned genes present in all genomes using MAFFT v7.526 (Katoh et al., 2002), and concatenated the sequences for phylogenetic inference. We inferred a maximum likelihood phylogenetic tree using IQ-TREE v2.3.4 (Minh et al., 2020) under the GTR + G + I model with 1,000 ultra-fast bootstraps. We annotated the trees using iTOL v6.9 (Letunic and Bork, 2024) and generated circular genome alignments using the Proksee server (Grant et al., 2023) with P01 as the reference sequence.

### Phenotypic characterization of phage P01

We characterized P01 for its morphological and phenotypic profiles, including host range, phage titer, optimal multiplicity of infection (MOI), stability at various pH values and temperatures, and *in vitro* phage bacteriolytic assay using methods as described previously (Fang et al., 2022; Yin and Zong, 2022). We observed the morphology of P01 using a JEM-1400PLUS transmission electron microscope (JEOL; Tokyo, Japan) at an accelerating voltage of 80 kV. We determined the host range of P01 against a collection of 32 CRKP strains belonging to 30 non-KL64 capsular types and 9 additional CRKP strains of KL64 (Table 1) using spot testing on double-layer agar plates. We tested the thermal stability of P01 by 1 h incubation of 10^8^ PFU per ml in TM Buffer at 4, 25, 37, 50, 60, or 70°C, followed by the double-layer agar method to check phage viability. Similarly, we also examined the stability of P01 at pH 1 to 13 in increments of 1.0 by 1 h incubation of 10^8^ PFU/ml in TM buffer at 37°C. We incubated P01 at 10^4^, 10^5^, 10^6^, 10^7^, or 10^8^ plague-forming units (PFU)/mL at 37°C for 3 h at a MOI of 0.01, 0.1, 1, or 10 to determine the optimal MOI.

**Table 1:** Host range of phage P01.

ST	Capsule	Strain	Accession no.	P01
45	KL62	020035	CP045988.1	−
37	KL158	030295	NWEP00000000	−
23	KL1	030925	CP073285.1	−
353	KL110	090235	JBEFBG000000000	−
15-1LV	KL28	090291	JBELVM000000000	−
107	KL103	090404	JBELVN000000000	−
3,393	KL35	090462	JBELVO000000000	−
497	KL16	090468	JBELVP000000000	−
708-1LV	KL116	090476	JBELVQ000000000	−
4,568	KL127	090481	JBELVR000000000	−
592	KL57	090515	CP073287.1	−
3,299	KL146	090526	JBELVS000000000	−
4,523	KL111	090529	JBEFBF000000000	−
3,242	KL15	090532	JBELVT000000000	−
872	KL153	090546	JBELVU000000000	−
4,023-2LV	KL13	090549	JBELVV000000000	−
1,537	KL24	090571	JBEFBE000000000	−
11	KL113	090608	JBELVW000000000	−
11	KL39	115057	JANHBK000000000	−
11	KL47	135025	JANHBV000000000	−
**11**	**KL64**	**135080**	**JANHBP000000000**	**+**
**11**	**KL64**	**140421**	**JBGTXX000000000**	**+**
**11**	**KL64**	**140159**	**JBGTXY000000000**	**+**
**11**	**KL64**	**140110**	**JBGTYA000000000**	**+**
**11**	**KL64**	**090525**	**JBGTYC000000000**	**+**
**11**	**KL64**	**115024**	**JBGTYD000000000**	**+**
**11**	**KL64**	**140011**	**JBGTYB000000000**	**+**
**11**	**KL64**	**140494**	**JBGTXV000000000**	**+**
**11**	**KL64**	**140490**	**JBGTXW000000000**	**+**
**11**	**KL64**	**140130**	**JBGTXZ000000000**	**+**
15	KL19	140101	JBELVX000000000	−
437-1LV	KL118	140125	JBEFBB000000000	−
11	KL21	140127	JANHBN000000000	−
101	KL106	140179	JBEFAZ000000000	−
11	KL25	140191	JANHBM000000000	−
412	KL57	140246	JAMWSC000000000	−
37	KL118	140249	JAMWRZ000000000	−
789	KL18	140308	JBELVY000000000	−
231	KL51	140398	JBELVZ000000000	−
17	KL112	140508	JBELWA000000000	−
211	KL17	140511	JBEFAW000000000	−
709	KL9	140529	JANHBL000000000	−

1LV, a novel ST with one allele difference; 2LV, a novel ST with two allele differences. Those that were lysed by P01 are in bold.

We investigated the *in vitro* bacteriolytic activity of P01 by adding phage at the optimal MOI (0.1) to the culture of strain 135080 in the pre-logarithmic phase, with an OD_600_ of 0.20, equivalent to approximately 10^8^ colony-forming units (CFU) per ml. We used the culture of strain 135080 without phages and LB broth without the bacterial strain as controls. We also determined the latent period and the burst size of P01 using the one-step growth experiments as described previously (Abedon and Katsaounis, 2018).

## Results

### Strain 135080 is an ST11-KL64 CRKP

The genome sequencing of strain 135080 generated 1.39 clean gigabases (Gb, 243× coverage), which were *de novo* assembled into 147 contigs (*N50*, 188,881 bp). This strain was identified as *K. pneumoniae* (*sensu stricto*) as it shares 98.86% ANI with the *K. pneumoniae* type strain ATCC 13883^T^ (Accession No. CP064368). This strain has *bla*_KPC-2_, a carbapenemase gene, and was indeed resistant to meropenem (MIC, 256 μg/mL) and imipenem (MIC, 128 μg/mL).

### Phage P01 lyses ST11-KL64 CRKP and represents a novel species of the genus *Taipeivirus* within the family *Ackermannviridae*

**Figure 1:**
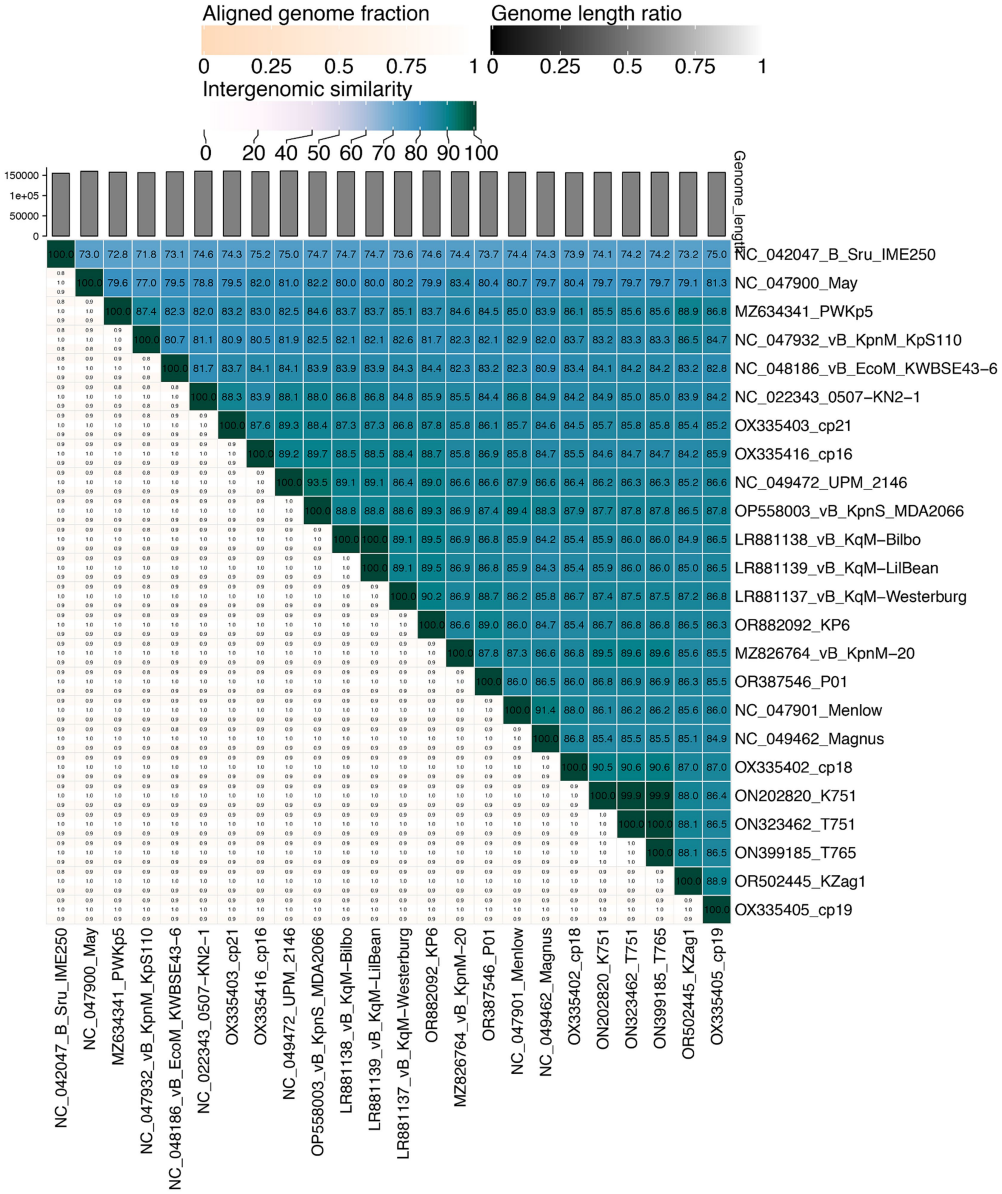
Mutual intergenomic similarities among *Taipeivirus* phages. This heatmap was generated using VIRIDIC (Moraru et al., 2020). Intergenomic similarities (%) are depicted in blue boxes and alignment genome fractions (0.8, 0.9, and 1.0) are present underneath. The phage names are shown with the accession numbers of their genome sequences.

**Figure 2:**
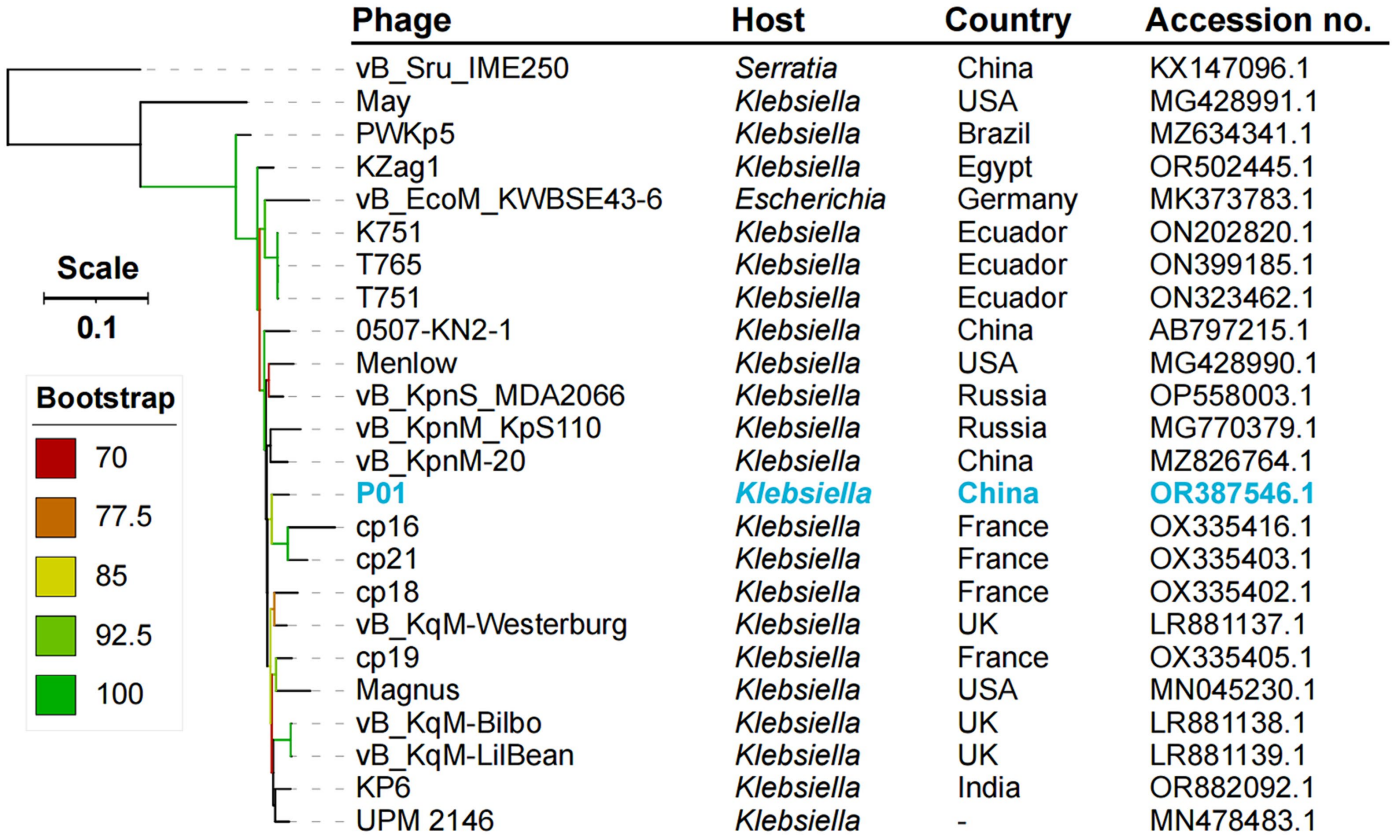
Phylogenetic tree of *Taipeivirus* phages based on the genome sequences. The maximum likelihood tree was constructed using IQ-TREE v2.3.4 (Minh et al., 2020) under the GTR + G + I model with 1,000 ultra-fast bootstraps and was annotated using iTOL v6.9 (Letunic and Bork, 2024). –, not available.

We obtained phage P01 from the river sample, which can lyse strain 135080. The genome sequencing of P01 produced 4,056,080 pairs of 150 bp reads, totaling 1.22 Gb. The complete genome of P01 was found to be 158,784 bp in length, with a 46.26% G + C content. It contained 237 genes (Supplementary material S1), along with four tRNA genes and one ncRNA. Notably, no known antimicrobial resistance or virulence genes were present in the genome of P01. In addition, no lysogeny genes were detected in P01, indicating its lytic nature. This was further supported by PhaTYP predictions, which classified P01 as a virulent phage with a full score. The abovementioned findings indicate that this phage is suitable for phage therapy (Suh et al., 2022). Using BLAST, the genome of P01 shows the highest overall DNA similarity (89.19%, identity × coverage) to *Klebsiella* phage vB_KqM-Westerburg (accession no. LR881137). This phage belongs to the genus *Taipeivirus* of the family *Ackermannviridae* within the order *Caudovirales* of the class *Caudoviricetes*. Notably, the 89.19% overall DNA similarity is below the 95% cutoff for defining species within the genus *Taipeivirus*. Consistent with the overall DNA similarity, the intergenomic similarity between P01 and all other *Taipeivirus* phages ranged from 73.7 to 89.0% (Figure 1). P01 is clustered within the *Taipeivirus* phages in maximum-likelihood phylogenetic trees based on the amino acid sequences of the DNA polymerase (Supplementary Figure S1) and the major capsid protein (Supplementary Figure S2). We also inferred a maximum-likelihood phylogenetic tree based on all orthologous genes, which also demonstrates the clustering of P01 with *Taipeivirus* phages (Figure 2). All the above analyses confirm its *Taipeivirus* membership, and according to the rule defined by the ICTV, P01 represents a novel species of the genus *Taipeivirus*.

### P01 has a distinct tail composition compared to other *Taipeivirus* phages

**Figure 3:**
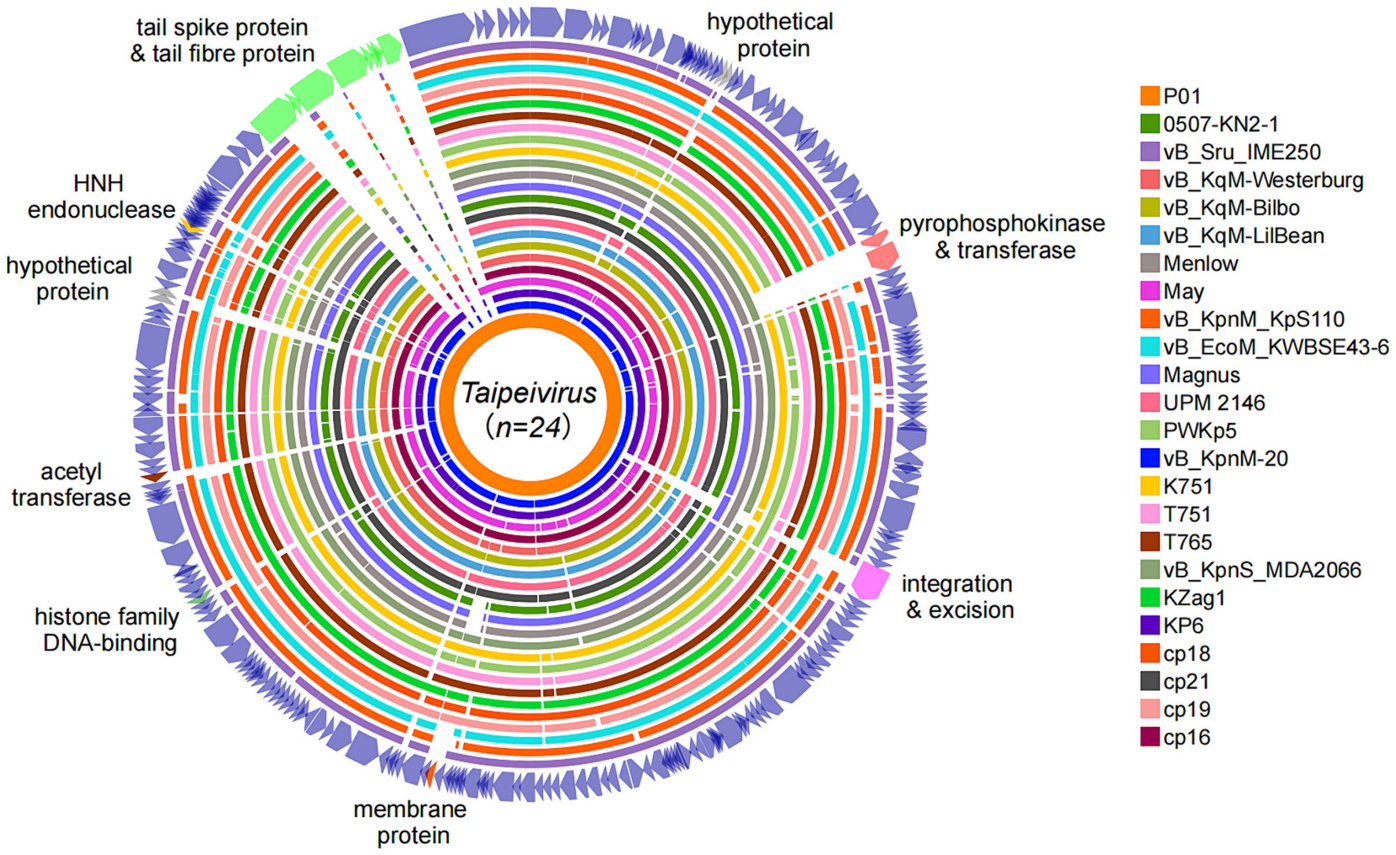
Genome alignments of *Taipeivirus* phages. Circular genome alignments were generated using the Proksee server (Grant et al., 2023) with P01 as the reference sequence and were annotated using iTOL v6.9 (Letunic and Bork, 2024).

Pairwise comparison of P01 with the 23 *Taipeivirus* genomes in GenBank revealed the overall similarity (identity × coverage), ranging from 73.61% (with phage vB_Sru_IME250, accession no. KX147096) to 89.19% (with vB_KqM-Westerburg, accession no. LR881137) (Supplementary material S2). Although the genome alignments showed a generally high average coverage of 88% against P01 (Supplementary material S1), we detected several genomic regions across the genome with varying degrees of similarity (Figure 3). These regions contained genes encoding proteins with diverse roles and functions. Specifically, we uncovered two adjacent genes, which are involved in ribose phosphate diphosphokinase activity when coupled with Mg^2+^, in 14 *Taipeivirus* phages, including P01 (Supplementary material S1). The two genes encode a pyrophosphokinase and a nicotinamide phosphoribosyl transferase, respectively, with an average nucleotide identity of 97.76 and 96.11% within the 14 *Taipeivirus* phages (Supplementary material S1). We also found an integrase gene in only six *Taipeivirus* phages including P01, sharing an average nucleotide identity of 98.57% (Supplementary material S1). Notably, we found that regions with genes classified as tail components were highly variable. Strikingly, P01 encodes two tail spike proteins. In contrast, other *Taipeivirus* phages typically lack genes encoding tail spikes, with only one such gene identified in phage May (accession no. MG428991) (Nguyen et al., 2019). Phage May shares an 85.26% nucleotide identity with one tail spike gene of P01 (Supplementary material S1). The other tail spike gene of P01 has no similar counterparts within *Taipeivirus*, and its encoded protein is most similar to a tail spike of an *Alcyoneusvirus* phage (named K64-1, accession no. NC_027399), sharing a 63.0% coverage and 56.25% amino acid identity. In addition, P01 also has two tail fiber protein genes, one of which is also present in all other 23 *Taipeivirus* phages with the highest nucleotide identity of 98.08% (Supplementary material S1). The other tail fiber protein gene has no similar counterpart within *Taipeivirus* but encodes a protein that is most similar to a tail fiber protein of *Alcyoneusvirus* phage K64-1 sharing a 100% coverage and 57.16% amino acid identity.

### P01 exhibits a *Myovirus*-like morphology, has a narrow host range, and is stable over a wide range of temperatures and pH values

**Figure 4:**
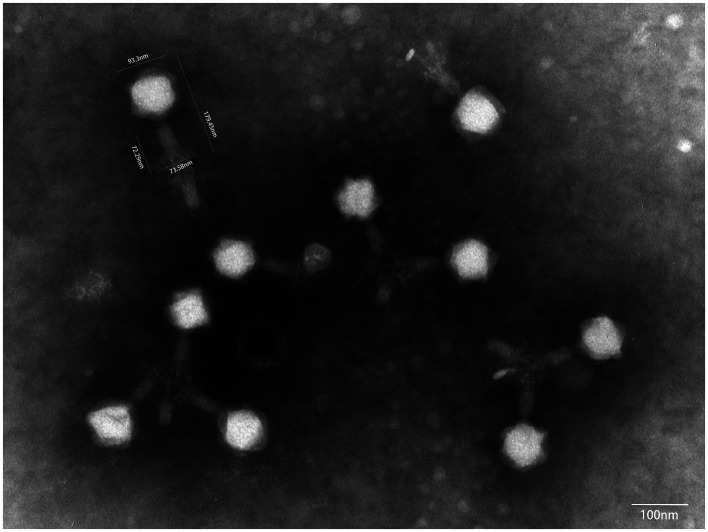
The transmission electron microscope image of P01. The image was obtained using a JEM-1400PLUS transmission electron microscope at an accelerating voltage of 80 kV.

**Figure 5:**
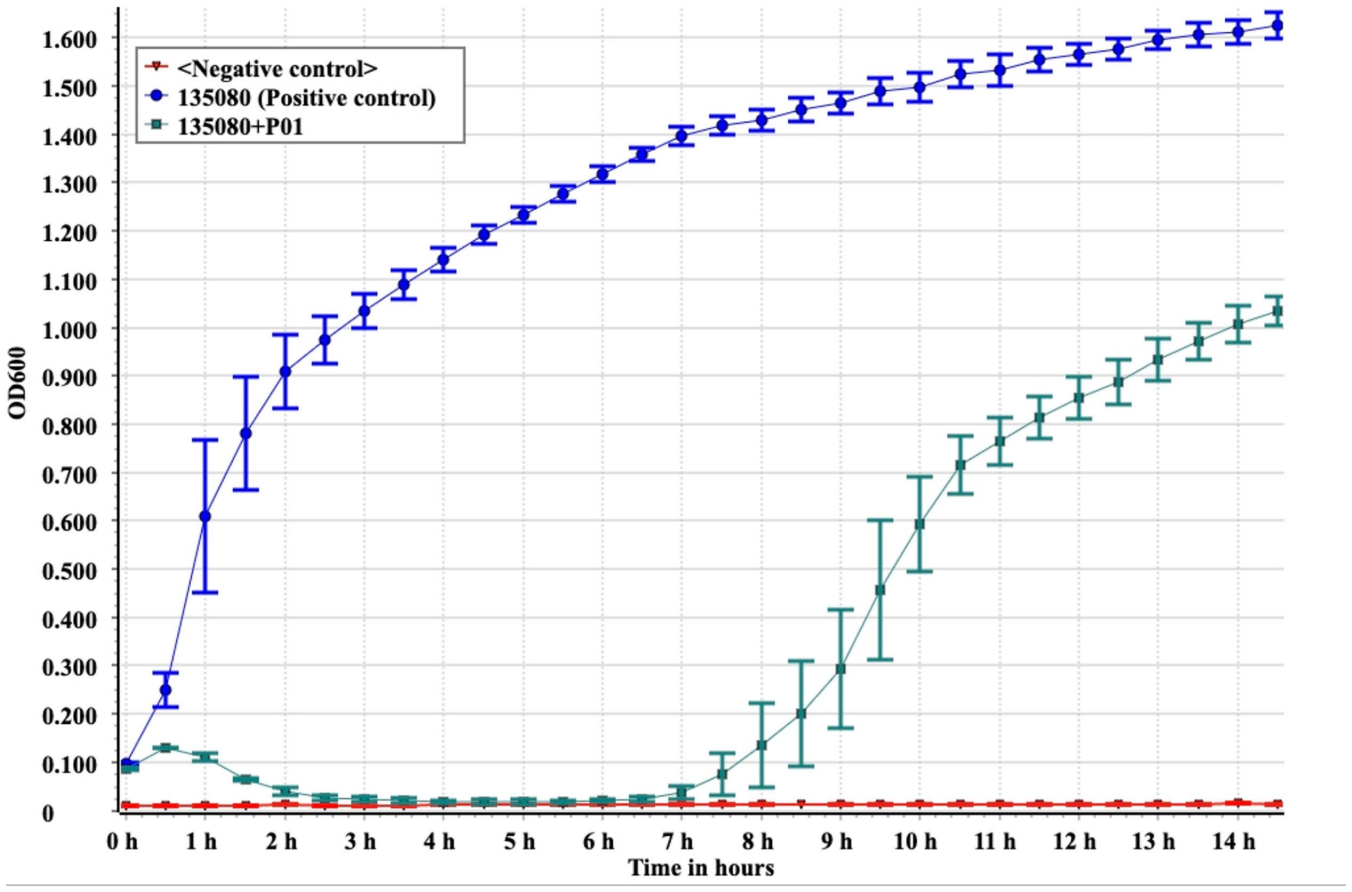
*In vitro* bactericidal assays of P01 against strain 135080. Assays were performed in triplicate. The median and standard deviation are shown. Negative control, LB broth.

Under a transmission electron microscope, P01 has a head of an isometric polyhedral structure, with approximately 93.3 nm in diameter and a long, rod-shaped, contractile tail of approximately 72.3 nm in length (Figure 4). Like other *Taipeivirus* phages, the appearance of P01 is consistent with *Myovirus*-like morphology (Townsend et al., 2021). In addition to strain 135080, P01 lysed the 9 additional CRKP strains of KL64, while it was unable to lyse non-ST11 strains or ST11 strains of a non-KL64 capsular type, exhibiting a narrow host range. P01 had a burst size of 143 progeny phages per infected bacterial cell and a latent period of 30 min. We found this phage to be stable in the temperature range of 4–50°C and at pH 3–12. We observed that the phage titer was all of 10^7^ PFU/mL at a MOI of 0.01, 0.1, 1, or 10, but was slightly lower (10^6^ PFU/mL) at a MOI of 100. P01 was able to inhibit the growth of strain 135080 for 6.5 h at a 0.1 MOI (Figure 5).

## Discussion

In this study, we recovered and characterized a novel phage with the ability to efficiently lyse the predominant CRKP type, ST11-KL64, in China. This phage carries no antimicrobial resistance nor virulence genes and is deemed suitable for phage therapy, thus expanding the biobank of phages viable against CRKP, a difficult-to-treat pathogen. In contrast to the majority of phages recovered so far against ST11-KL64 CRKP, which are members of the genus *Przondovirus* within the family *Autographiviridae*, P01 belongs to a novel species of the genus *Taipeivirus* within the family *Ackermannviridae*. This suggests that phages from distinct taxonomic positions can target a common bacterial host species.

The genus *Taipeivirus* was first proposed in 2019 (Korf et al., 2019), while the isolation of the first *Taipeivirus* phage can be traced back to 2013 (Hsu et al., 2013). As mentioned above, in addition to P01, there are 23 identified *Taipeivirus* phages: 21 isolated using *Klebsiella* host strains, one with *E. coli*, and one with *S. rubidaea*. This indicates that *Taipeivirus* phages are viruses that infect Enterobacterales. *Taipeivirus* phages typically exhibit a narrow host range with the ability to attack a certain species or *Klebsiella* strains of one or more specific capsular types (Acevedo Ugarriza et al., 2019; Korf et al., 2019; Martins et al., 2021, 2022; Lourenco et al., 2023; Tisalema-Guanopatin et al., 2023; Wu et al., 2023). For instance, phage vB_EcoM_KWBSE43-6, which was recovered using an *E. coli* strain, cannot infect *K. pneumoniae* and *Klebsiella oxytoca* (Korf et al., 2019). Furthermore, phages vB_KqM-LilBean, vB_KqM-Bilbo, and vB_KqM-Westerburg were isolated using *Klebsiella* strains of the KL35 capsular type (Townsend et al., 2021), while P01 cannot lyse KL35 *Klebsiella* (Table 1). In addition, vB_KpnM-20 cannot lyse KL64 *Klebsiella* (Wu et al., 2023) in contrast to P01. Capsular-specific activity indicates that capsules provide the receptor for *Taipeivirus* phages. The phage host range is determined by the receptor-binding proteins that form the baseplate-attached tail spike or tail fiber proteins (Dunne et al., 2018; Yehl et al., 2019). *Taipeivirus* phages contain one to four (median, three) tail fiber proteins but typically lack tail spike proteins (Supplementary material S1). Compared to other *Taipeivirus* phages, P01 has a distinct composition comprising two tail spike proteins plus two tail fiber proteins. Notably, one tail spike protein and one tail fiber protein of P01 are not present in other *Taipeivirus* phages, but both are most similar to the counterparts of an *Alcyoneusvirus* phage. *Alcyoneusvirus* is an independent genus within the class *Caudoviricetes*, but without being assigned to a specific family or an order.^5^ It is likely that the two P01-unique tail protein-encoding genes were acquired from other, as yet identified, non-*Ackermannviridae* phages within the class *Caudoviricetes*. Nevertheless, the highly variable tail composition provides the flexibility for *Taipeivirus* phages to adapt to different Enterobacterales strains. The differences between the tail spike and fiber proteins highlight that phage tails are the key evolutionary hotspots in the arms race of phages with their bacterial hosts.

## Data Availability

The draft genome sequence of strain 135080 and the complete sequence of phage P01 have been deposited in GenBank under accession numbers JANHBP000000000 and OR387546, respectively.

## Footnotes

^1^ https://pubmlst.org/organisms/klebsiella-aerogenes

^2^ https://blast.ncbi.nlm.nih.gov/Blast.cgi

^3^ https://ictv.global/taxonomy/taxondetails?taxnode_id=202308036&taxon_name=Taipeivirus

^4^ https://github.com/gbouras13/phold

^5^ https://ictv.global/taxonomy/taxondetails?taxnode_id=202106653
