# Supplementary material for: Ackermannviridae bacteriophage against carbapenem-resistant Klebsiella pneumoniae of capsular type 64
Source: Front Microbiol. 2024 Sep 23;15:1462459. doi: 10.3389/fmicb.2024.1462459 (PMC11456439; doi:10.3389/fmicb.2024.1462459)

**Supplementary materials**

**Supplementary Figure S1.** The maximum-likelihood phylogenetic tree based on amino acid sequences of the DNA polymerase of all *Taipeivirus* phages.


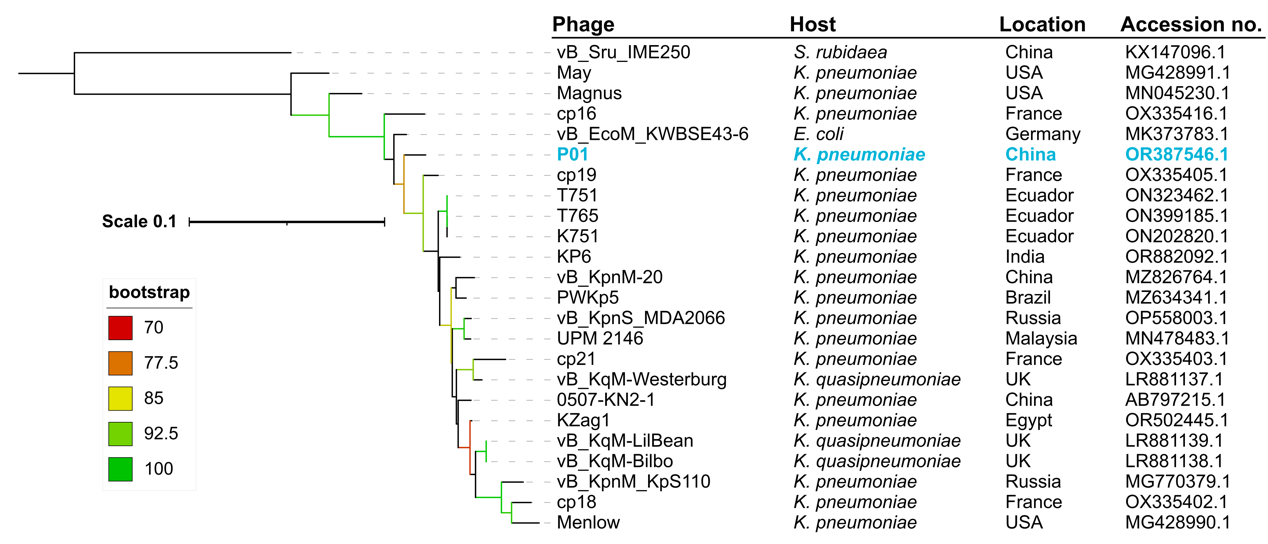


**Supplementary Figure S2.** The maximum-likelihood phylogenetic tree based on amino acid sequences of the major capsid protein of all *Taipeivirus* phages.


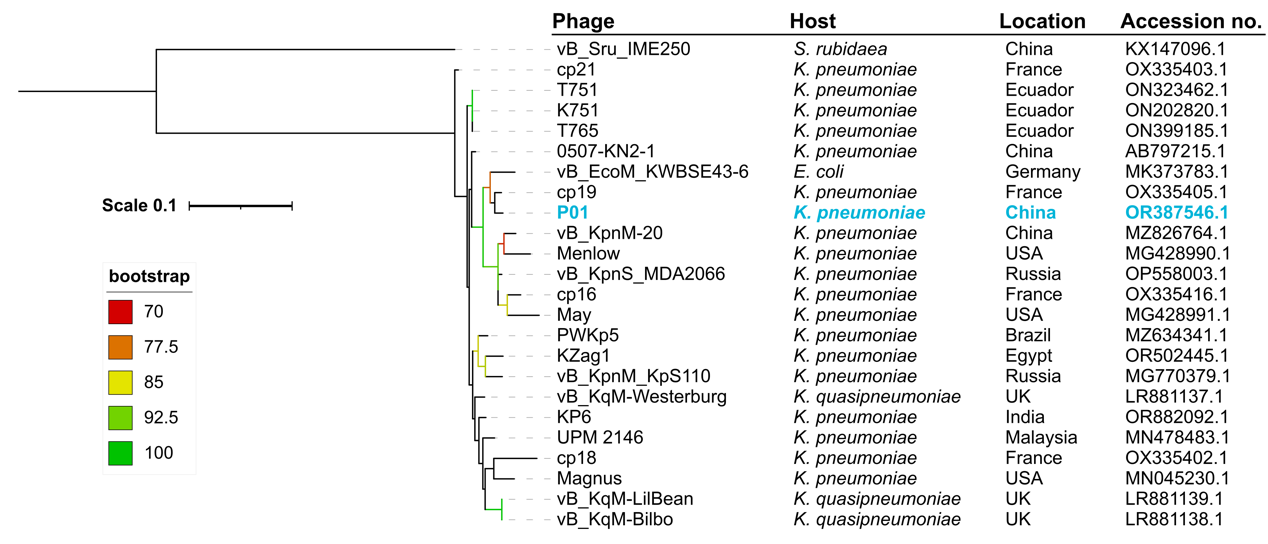

Supplement: Supplementary file 2 [file Data_Sheet_1.docx]
